# Bioremediation of Automotive Residual Oil-Contaminated Soils by Biostimulation with Enzymes, Surfactant, and Vermicompost

**DOI:** 10.3390/ijerph20166600

**Published:** 2023-08-18

**Authors:** Omar Sánchez Mata, Miguel Mauricio Aguilera Flores, Brenda Gabriela Ureño García, Verónica Ávila Vázquez, Emmanuel Cabañas García, Efrén Alejandro Franco Villegas

**Affiliations:** 1 Interdisciplinary Professional Unit of Engineering, Campus Zacatecas, Instituto Politécnico Nacional, Blvd. del Bote 202 Cerro del Gato Ejido La Escondida, Col. Ciudad Administrativa, Zacatecas 98160, Mexicovavila@ipn.mx (V.Á.V.);; 2 Scientific and Technological Studies Center No. 18, Instituto Politécnico Nacional, Blvd. del Bote 202 Cerro del Gato Ejido La Escondida, Col. Ciudad Administrativa, Zacatecas 98160, Mexico

**Keywords:** automotive residual oil-contaminated soils, biostimulation, enzymatic treatment, soil bioremediation, surfactant, vermicompost

## Abstract

Contamination of soils by automotive residual oil represents a global environmental problem. Bioremediation is the technology most suitable to remove this contaminant from the medium. Therefore, this work aimed to evaluate the effectiveness of bioremediation of automotive residual oil-contaminated soils by biostimulation with enzymes, surfactant, and vermicompost. The bioremediation efficiency was examined using a factorial design of 2^4^ to determine the effect of the time, pH and temperature conditions, biostimulation with enzyme-vermicompost, and biostimulation with enzyme-surfactant. Enzymes obtained from *Ricinus communis* L. seeds, commercial vermicompost, and Triton X-100 were used. Results showed that the highest removal efficiency (99.9%) was achieved at 49 days, with a pH of 4.5, temperature of 37 °C, and using biostimulation with enzyme-vermicompost (3% *w*/*v*–5% *w*/*w*). The addition of surfactant was not significant in increasing the removal efficiency. Therefore, the results provide adequate conditions to bioremediate automotive residual oil-contaminated soils by biostimulation using enzymes supported with vermicompost.

## 1. Introduction

Petroleum hydrocarbons (oil and petroleum products such as diesel, gasoline, and automotive oil) represent the most frequent and widespread environmental contaminant [1,2]. Their introduction into a pristine environment changes nature, causing reduced ecosystem functionality and increased human health risks [1]. These hydrocarbons include alkanes, aromatic compounds, nitrogen, and sulfur-oxygen-containing compounds. The aromatic fraction includes polycyclic aromatic hydrocarbons, listed as priority contaminants since they are toxic, carcinogenic, mutagenic, and environmentally recalcitrant [3]. Such is the case of the automotive residual oil that contains benzene and heavy metals (lead, arsenic, zinc, and cadmium), becoming a potential contaminant when this waste is inadequately disposed [4]. Hence, petroleum hydrocarbon-contaminated soils are a global environmental problem [5].

Bioremediation is recognized as the most environmentally friendly remediation technology for petroleum hydrocarbon removal from an environment since it does not require rigorous mechanical, chemical, and expensive interventions [1]. This procedure can be performed ex situ when the contaminated soil is removed from the subsoil to treat it at the same or at another location, or in situ when the contaminants are eliminated directly from the contaminated soil. Generally, bioaugmentation and biostimulation are the most common techniques worldwide for the bioremediation of petroleum hydrocarbon-contaminated soils performed in situ or ex situ. Bioaugmentation is the intentional supplementation of contaminant-specific degrading microorganisms in contaminated soil to increase the biodegradation rate [6,7,8]. Biostimulation involves the addition of nutrients or fertilizers (amendments) to stimulate the growth and the metabolic and degradative activities of microorganisms present in contaminated soils (indigenous microbes) since there is generally a scarcity of nitrogen and phosphorus in the contaminated sites, limiting the availability of these nutrients for the survival of the contaminant-degrading microorganisms [8].

Although bioaugmentation has shown more favorable results than biostimulation, it has also been reported that biostimulation shows better results than bioaugmentation when performed using ideal conditions of temperature, pH, and concentration, among other parameters [4,8,9]. Organic and inorganic fertilizers are nutrients used in the biostimulation technique. However, organic fertilizers are more effective than inorganic fertilizers since they increase organic matter content and microbial activity in contaminated soil [8]. One of the organic fertilizers used in this technique is vermicompost. This product is generated from the biological decomposition of organic waste through the combined action of earthworms and microorganisms [10]. Therefore, vermicompost is a rich organic fertilizer in nutrients (nitrogen, potassium, phosphorus), which enhances microbial activity, nutrient recycling, and soil quality [11], becoming an effective biostimulant applied in hydrocarbon-contaminated soil remediation [10].

On the other hand, surfactants have been used in solutions to remove petroleum hydrocarbons from soil mainly by the soil flushing technique [12]. The surfactants increase the mobility and solubility of these contaminants by emulsification. Therefore, petroleum hydrocarbons become more available to be degraded by microorganisms [13]. The best surfactants are anionic and non-ionic [12]. Triton X-100 is a non-toxic and non-ionic surfactant that could be used in the biostimulation technique to promote the availability of these contaminants and increase the biodegradation rate [12,13].

In addition, different enzymes (mono- or dioxygenases, halogenases, peroxidases, phosphotriesterases, hydrolases, transferases, and oxidoreductases) obtained from various species of microorganisms and plants have been used for the bioremediation of contaminated soils. The specificity of the enzymes makes them act on distinct molecules with similar structures. Likewise, their efficiency and stability can be improved for exclusive conditions or specific substrates. They could be used in isolation or wholly in the bioremediation process, adding to the contaminated area [14]. Likewise, enzymatic bioremediation could be in situ or ex situ. Although in situ is less expensive, ex situ is feasible for highly contaminated soils and when it is necessary to provide conditions (pH and temperature) for the enzyme’s activity and a fast remediation action [14,15].

Lipase, esterases, cutinase, nitrilases, aminohydrolases, and organophosphorus hydrolase are the hydrolase enzymes used in the organic compounds-contaminated soil bioremediation [14,15]. Various authors have reported the application of enzymes to remove organic contaminants from soil, such as phenolic compounds and polycyclic aromatic hydrocarbons [16,17]; benzene, toluene, ethylbenzene, and xylene (BTEX) [18]; and petroleum hydrocarbons [19,20].

Aguilera-Flores et al. [20] used enzymes obtained from *Ricinus communis* L seeds to catalyze the biodegradation of automotive residual oil in the soil. They examined the remediation process under two conditions. The first one was at room temperature and without the modification of soil pH. The second one was at 37 °C and a soil pH of 4.5. This last condition was used since the *Ricinus communis* L. enzymes show the highest catalytic activity. After seven weeks of treatment, automotive residual oil removal percentages of 14% and 94% were obtained, respectively, demonstrating that the pH and temperature conditions are essential for enzyme function in the remediation process, being effective as an ex situ treatment. However, both remediation assays could be improved with surfactants or amendments.

Therefore, this study aimed to evaluate the effectiveness of bioremediation of automotive residual oil-contaminated soils by biostimulation with enzymes, surfactant, and vermicompost. The bioremediation efficiency was examined using a factorial design of 2^4^ to determine the effect of the time as a numeric factor, and pH and temperature conditions, biostimulation with enzyme and vermicompost, and biosimulation with enzyme and surfactant as categoric factors. The results provide information on the adequate conditions to bioremediate automotive residual oil-contaminated soils by biostimulation using enzymes supported with surfactant and vermicompost.

## 2. Materials and Methods

### 2.1. Obtaining and Treatment of Feedstocks 

*Ricinus communis* L. fruits were collected on public land where it grows wild in the city of Jerez (Zacatecas, Mexico), located at the latitude of 22°28′30″ N and longitude of 102°59′08″ W. This plant is considered an invasive exotic species in Mexico and other countries [21,22,23]. Therefore, a permit or regulation was not required for its collection. The *Ricinus communis* L. fruits were cut with pruning shears, wrapped in black plastic, and sun-dried until the seeds were exposed. The seeds were put in a Ziploc bag and stored at 4 °C until their use.

The enzymes from *Ricinus communis* L. seeds were obtained following the methodology described by Aguilera-Flores et al. [20], based on Avelar et al. [24]. These enzymes were stored and kept sterile at 4 °C until used in the bioremediation tests.

Fifteen simple soil samples were taken on a terrain of 0.055 hectares in Zacatecas (Zacatecas, Mexico), located at the latitude of 22°44′52″ N and longitude of 102°35′13″ W, ensuring that they were free of automotive residual oil and other hydrocarbons. Sampling was performed in a random zig-zag pattern, taking 1 kg of soil sample at each point. These soil samples were mixed to form a composite soil sample of 15 kg. This composite soil sample was sun-dried, sieved with a 2 mm mesh, and stored until its use at room temperature in a Ziploc bag [25].

Triton X-100 surfactant (Golden Bell brand), commercial vermicompost (Vita Organic brand), and automotive residual oil (provided by a mechanical workshop) were used in the bioremediation tests described in Section 2.3.

### 2.2. Soil and Vermicompost Physical-Chemical Characterization

The physical-chemical properties of the soil such as the pH, bulk density, moisture retention, organic matter content, inorganic nitrogen content, and texture were analyzed according to the methods AS-02, AS-03, AS-06, AS-07, AS-08, and AS-09, respectively, cited by the Mexican standard NOM-021-SEMARNAT-2000 [26].

The physical-chemical characterization of the commercial vermicompost revealed the following properties: pH 7.9, electrical conductivity 6180 µS/cm, organic matter content 155.7 g/kg, inorganic nitrogen content 8.9 g/kg, C/N ratio 17.3, and phosphorus content 0.027 g/kg. The contents of organic matter, inorganic nitrogen, and phosphorus were relevant to determine the amount of vermicompost to add to the corresponding bioremediation tests.

### 2.3. Bioremediation Tests

The soil contamination was performed by homogenizing the automotive residual oil (10,000 mg) and soil (1 kg) using a V cone blender CEN-MKII-11 (Armfield, Ringwood, United Kingdom) at 40 rpm for 30 min. A concentration of 10,000 mg of automotive residual oil per kg of soil was induced for each test. After homogenization, the sample was transferred to plastic trays with dimensions of 30 cm × 45 cm, adapting the conditions shown for each bioremediation test in Table 1.

The bioremediation tests were performed using a factorial design of 2^4^ with two replicates. This experimental design was selected to determine the effect of a numeric factor (time) and three categoric factors (pH and temperature conditions, biostimulation with enzyme and vermicompost, and biostimulation with enzyme and surfactant).

The low and high levels of 14 and 49 days, respectively, were used for the time factor. These values were established based on the study of Aguilera-Flores et al. [20], where they observed that the automotive residual oil remotion began after 14 days, achieving almost complete removal at 49 days, using the enzymes obtained from *Ricinus communis* L. seeds.

The low and high levels of pH and temperature conditions were defined as a function of the enzyme activity. The enzymes obtained from *Ricinus communis* L. seeds show the highest enzymatic activity at a pH of 4.5 and a temperature of 37 °C [27]. This condition was used for the low level of this factor and was named ideal conditions. The soil pH was adjusted with a solution of sulfuric acid (H_2_SO_4_) 0.5 M, gradually adding volumes of 0.5, 1, and 2 mL at a time and homogenizing the soil with each solution addition until a pH of 4.5. These bioremediation tests were incubated to keep the temperature at 37 °C in an Incubator Series B (BINDER GmbH, Tuttlingen, Alemania). The high level was defined as ambient conditions of pH and temperature, where the soil pH was not modified, and the bioremediation tests were performed at room temperature.

The low and high levels of the biostimulation with enzyme and vermicompost were defined in function of the absence or presence of vermicompost, respectively. The nutrient ratios (organic matter, inorganic nitrogen, C/N ratio, and phosphorus) of different hydrocarbon bioremediation tests performed by other authors were taken as a reference [28,29,30]. Therefore, 50 g of vermicompost per kg of soil was added to obtain a concentration of 5% *w*/*w*. Likewise, the absence or presence of surfactant was defined as the low or high level for the biostimulation with enzyme and surfactant, respectively. Based on Cecotti et al. [31], 26 g of Triton X-100 surfactant per kg of soil was added to the corresponding bioremediation tests. The enzymes were added to all the tests using 100 mL of a 3% *w*/*v* solution.

The experimental design consisted of 32 tests (Table 1), which were performed at the laboratory in random order. The percentages of automotive residual oil removal were taken as the response in the experimental design. Subsequently, a statistical analysis (ANOVA) was carried out, described in Section 2.5.

### 2.4. Calculation of the Percentage of Automotive Residual Oil Removal

Soil sub-samples of approximately 10 g were removed from bioremediation tests once the time was up. Then, 10 g anhydrous sodium sulfate was added to the soil sub-samples to be transferred into an extraction cartridge of 20-micron filter paper. The remaining amount of automotive residual oil in each test was determined by the Soxhlet extraction method using 120 mL per sub-sample of dichloromethane as the solvent, according to the methodology EPA 9071B stipulated by Mexican standard NMX-AA-134-SCFI-2006 guidelines [32], with modifications. The extraction process was performed at 40 °C for 240 min. The measurement of the weight of the extractable material with dichloromethane consisted of distilling in a simple distillation device at 40 °C. The solvent was recovered, and the flask was transferred to a desiccator for 30 min, where the flask was weighed, recording its weight ($C_{i}$). Previously, the weight value of the uncontaminated soil ($M_{e}$) was recorded using the same Soxhlet extraction operating conditions described above. After a relationship between and with the difference of weights against the initial weight of the residual automotive oil of contaminated soil ($C_{0}$) was made, obtaining the removal percentage of automotive residual oil by Equation (1).

(1) $$Removal\;of\;automotive\;residual\;oil\;\left(\%\right)=\frac{C_{0}-\left(C_{i}-M_{e}\right)}{C_{0}}\times 100$$

where $C_{0}$ is the mg initial of automotive residual oil per mg of contaminated soil, $C_{i}$ is the mg of automotive residual oil extracted per mg of contaminated soil, and $M_{e}$ is the mg of removable material in non-contaminated soil per mg of this soil.

### 2.5. Statistical Analysis

An analysis of variance (ANOVA) was performed using Design-Expert^®^ software (trial version 12) (Stat-Ease, Inc., Minneapolis, MN, USA). The ANOVA considered the main effects of the model factors. The evaluated factors were Factor A: time, factor B: pH and temperature conditions, factor C: biostimulation with enzyme and vermicompost, and factor D: biostimulation with enzyme and surfactant. The significance of the model was determined at a *p*-value < 0.05. Likewise, an analysis of the significance of each factor was performed considering the *p*-value.

### 2.6. Fourier Transform Infrared (FTIR) Spectroscopy Analysis 

FTIR analysis was performed according to the methodology EPA 418.1 with modifications described by Schwartz et al. [33]. The extracts obtained by Soxhlet extraction in the last analysis of the test numbers 5, 7, 14, and 15 and a blank soil sample without contamination with automotive residual oil were diluted with 30 mL dichloromethane. This mixture was kept in a sealed glass vial with a PTFE cap and placed in a sonic bath to hasten the extraction process for 30 min. Five silica gel beads were added to the mixture to absorb any polar hydrocarbon. The product was recuperated by filtration and measured in an FTIR spectrometer (Bruker, Billerica, MA, USA). The spectra were collected in 32 scans at 4 cm^−1^ in the mid-IR range of 4000–400 cm^−1^ with automatic signal gain and rationed against a background spectrum recorded from the clean empty cell at 25 °C. Spectral data analysis was performed using the OPUS 3.0 data collection software program (Bruker, Billerica, MA, USA). Test numbers 5, 7, 14, and 15 were selected based on the conditions and percentages of automotive residual oil removal obtained, which allowed a better discussion.

## 3. Results and Discussion

### 3.1. Influence of the Physical-Chemical Parameters of the Soil in the Bioremediation Tests

Table 2 shows the results of the physical-chemical characterization of the soil sampled and used in the bioremediation tests.

The pH plays an essential role in the degradation of hydrocarbons in the soil, showing positive correlations in increasing the rate of degradation of these pollutants and alternating the conditions of microorganisms and the activity of enzymes capable of degrading hydrocarbons in the soil. The optimal pH ranges between 5 and 8 [34]. However, it has been shown that the optimum pH for the hydrocarbon hydrolysis catalysis for the *Ricinus communis* L. enzymes is 4.5 [27]. Aguilera-Flores et al. [20] reported favorable results in the remediation of hydrocarbon-contaminated soils, modifying the soil pH to 4.5 to increase the catalytic activity of *Ricinus communis* L. enzymes. Considering the results provided by the authors mentioned [20,27], the pH was adjusted to 4.5 for ideal conditions tests, and the value shown in the characterization of the soil sampled (Table 2) was used for the ambient conditions tests.

In the same way, the nutrients also play a significant role in the bioremediation of hydrocarbon-contaminated soils since soils with autochthonous microorganisms capable of degrading hydrocarbons will be affected if there is a high concentration of hydrocarbons and they do not have enough nutrients to stimulate their growth. Consequently, it has been reported that macronutrients such as carbon, nitrogen, and phosphorus stimulate the microorganisms’ growth, accelerating hydrocarbon degradation [35]. Various authors have found that C/N ratios between 16 and 36 increase hydrocarbon degradation in bioremediation processes [28,29,30]. And favorable results have been obtained in hydrocarbon-contaminated soils bioremediation when biostimulating with vermicompost [36]. Therefore, this study used vermicompost to biostimulate the bioremediation process, adjusting the C/N ratio to 17.3 by adding 5% *w*/*w* since 57.1 was obtained from the physical-chemical characterization of the soil shown in Table 2.

The texture is a parameter that determines the type of soil according to the predominant particles found in it. The soil of this study was classified as sandy loam based on the sand, clay, and silt content shown in Table 2 and that reported by Nachtergaele et al. [37]. The soil texture and apparent density impact the mobility of hydrocarbons and nutrients, the amount of water that can be retained, and the oxygen available for the hydrocarbon-degrading microorganisms [38]. It has been reported that hydrocarbon degradation is favored in the textural classes of sandy-type soil since sandy soils are more porous than clays and, therefore, there is a higher transfer of oxygen necessary for the hydrocarbon-degrading microorganisms [39]. Furthermore, the pore size also affects the microorganisms’ growth since it has been reported that pores smaller than 3 μm are not accessible to bacteria [39]. Hence, the degradation of hydrocarbons is more efficient in sandy-type soils than in clayey-type soils. Likewise, sandy-type soils show a better advantage in bioremediation processes since clayey-type soils tend to perform strong adsorption of contaminants on the surface of the clays, reducing microbial biodegradation [39]. Therefore, the bioremediation tests were favored by the textural class of the soil used in this study.

### 3.2. Results of the Automotive Residual Oil Removal

Table 3 shows the results of the automotive residual oil removal obtained for each bioremediation test performed based on the experimental design.

Tests 3, 4, 7, and 8 used *Ricinus communis* L. enzymes at ambient conditions without biostimulation with vermicompost and or surfactant. The efficiency of the percentage of automotive residual oil removal was increased two times at 49 days compared to day 14 (Table 3). In contrast, tests 1, 2, 5, and 6 were performed at ideal conditions, increasing the removal percentage five times. These results agree with those of Aguilera-Flores et al. [20], who reported automotive residual oil removal efficiencies of 14.11% and 94.26% after seven weeks of treatment using *Ricinus communis* L. enzymes at ambient and ideal conditions, respectively.

When the vermicompost was the amendment used in the biostimulation (tests 9–16), automotive residual oil removal percentages increased 4.4 and 1.1 times at ambient and ideal conditions in 49 days, respectively. Almost complete removal was achieved at ideal conditions, and the removal efficiency was enhanced at ambient conditions. Therefore, adding nutrients with vermicompost stimulates the automotive residual oil biodegradation. It must be noted that the highest removal percentages were obtained in tests 13 and 14, corresponding to the tests where the biostimulation was performed with enzyme and vermicompost at ideal conditions.

On the other hand, when the tests were performed by biostimulation with enzyme and surfactant (tests 17–24), the percentages of automotive residual oil removal decreased 1.1 times for both conditions regarding tests 9–16. Likewise, these percentages decreased (1.1. times) at ideal conditions regarding tests 5 and 6 for 49 days. However, these values were increased 4.2 times at ambient conditions regarding tests 7 and 8 for 49 days. Therefore, adding surfactant enhanced the tests performed at ambient conditions, although its efficiency is lower than when vermicompost is used. However, the surfactant reduces the removal efficiency at ideal conditions since the enzymes’ catalytic activity could be affected.

Although a proper behavior has not yet been identified, studies of the inhibition or enhancement of the catalytic activity of enzymes at solid/liquid and liquid/liquid interfaces have focused on explaining the relationship between the structure of the enzyme and the environment where it works [40]. It has been observed that enzymes can catalyze a wide range of reactions at the interface of the aqueous and organic phases. However, it has also been noted that non-ionic surfactant concentration impacts the catalytic activity of the enzymes, where low concentrations of non-ionic surfactants increase catalysis while increasing the concentration of non-ionic surfactants inhibits enzymatic activity. This ratio of catalytic activity and non-ionic surfactants has a mixed effect because some surfactants, such as Tween and Triton, often increase the catalytic activity of some enzymes but can also inhibit them [41]. However, because Triton X-100 is a non-ionic surfactant [42], no literature relationship has been found regarding the inhibition or increase in the catalytic activity of enzymes containing the species *Ricinus communis* L. and Triton X-100 used in the remediation of hydrocarbon-contaminated soils. The statistical analysis of the present study suggests that using Triton X-100 in conjunction with *Ricinus communis* L. enzymes in the soil inhibits enzymatic activity, as is shown in Section 3.3 for factor D, which corresponds to biostimulation with enzyme and surfactant, and wherein the ANOVA evaluation of this factor showed that it was not significant in the removal of automotive residual oil.

Finally, when the tests were performed by biostimulation with enzyme, vermicompost, and surfactant (tests 25–32), the removal efficiencies were like those achieved in the biostimulation with enzyme and vermicompost (tests 9–16). The interaction that the catalytic activity has with the surfactant concentration used in the present study is not known since, as was mentioned, the enzymatic activity could be inhibited when used at a high surfactant concentration, and it could increase at low concentrations [40,41,42]. This effect could be present in the bioremediation tests performed by adding surfactant Triton X-100. However, there are no previous studies where the catalytic activity of the *Ricinus communis* L. enzymes was affected by the interaction of the soil-Triton X-100 interface. Therefore, although this study did not evaluate this interaction, it can lay the foundations for future studies. Hence, it can be inferred that the surfactant does not have a significant effect on the bioremediation process. However, this assumption was confirmed by statistical analysis.

### 3.3. Results of the Analysis of Variance ANOVA

Table 4 shows the results of the analysis of variance ANOVA performed from the experimental design of 2^4^, considering the automotive residual oil removal percentage as the response. It can be noted that the model and the factors A, B, and C were significant (Table 4) since a *p*-value < 0.1 was considered significant, while a *p*-value > 0.1 was not significant [43]. The factor D was not significant (*p*-value > 0.01). Therefore, the surfactant does not affect the response when used in the biostimulation. This result coincides with the automotive residual oil removal percentages where the tests performed with surfactant were affected, obtaining lower removal percentages than in the tests where vermicompost without surfactant was employed (tests 17–32).

On the other hand, the time (factor A) and the pH and temperature conditions (factor B) were the factors that most affected the automotive residual oil removal percentage with a *p*-value < 0.1 (Table 4). The highest removal percentages were achieved when the bioremediation tests were performed with a soil pH of 4.5 and a temperature of 37 °C for 49 days. Therefore, conditioning these parameters (pH and temperature) so that *Ricinus communis* L. enzymes show their highest catalytic activity is essential to obtain effective contaminant removal.

Following these factors (A and B), factor C (biostimulation with enzymes and vermicompost) affects the response with a *p*-value < 0.1 (Table 4). Hence, using vermicompost favored the automotive residual oil removal in the bioremediation tests. This condition can be confirmed by the results shown in Table 3, in which the highest removal percentages were obtained in tests 13 and 14 (Table 3) using biostimulation with enzymes and vermicompost (factor C) at ideal conditions (factor B, low-level) for 49 days (factor A, high-level). Therefore, adding vermicompost as an amendment increased the automotive residual oil removal percentage from 94.38% (tests 5 and 6) to 99.90% (tests 13 and 14) regarding the biostimulation only with *Ricinus communis* L. enzymes at ideal conditions (soil pH of 4.5, 37 °C).

The experimental design used in this study is limited since it does not allow evaluation of the interactions between factors or upper levels (quadratic and cubic effects). Therefore, the factors of this work should be studied using other experimental designs that include interactions and non-linear effects. A precedent on the optimization of the bioremediation process by biostimulating with *Ricinus communis* L. enzymes and vermicompost was not found in the literature. Thus, this investigation opens a new panorama of possible optimization in the bioremediation of automotive residual oil-contaminated soils by biostimulation with vermicompost and *Ricinus communis* L. enzymes.

### 3.4. Results of the Analysis of FTIR

**Figure 1:**
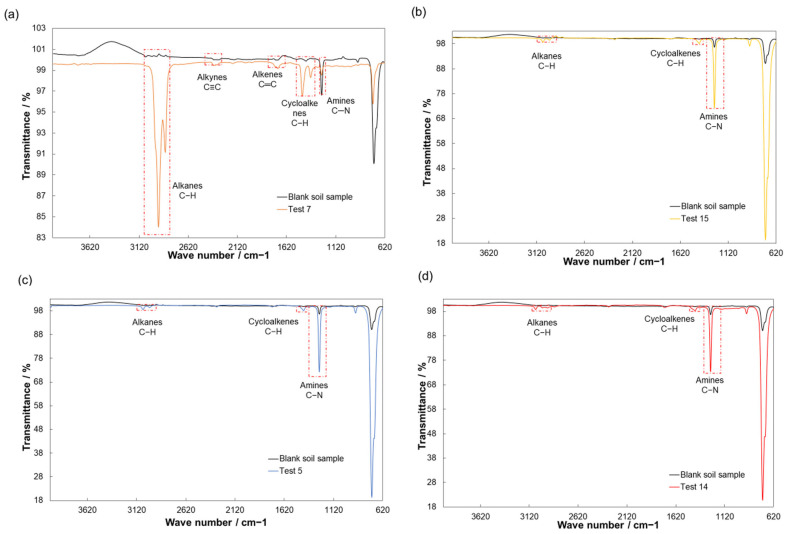
FTIR analysis of automotive residual oil-contaminated soil and its comparison with uncontaminated soil. (**a**) Biostimulation with *Ricinus communis* L. enzymes at ambient conditions for 49 days (test 7), (**b**) biostimulation with *Ricinus communis* L. enzymes and vermicompost at ambient conditions for 49 days (test 15), (**c**) biostimulation with *Ricinus communis* L. enzymes at ideal conditions for 49 days (test 5), (**d**) biostimulation with *Ricinus communis* L. enzymes and vermicompost at ideal conditions for 49 days (test 14).

The responses with the lowest (ideal conditions) and highest (ambient conditions) levels for factor B were considered. Likewise, the tests performed by biostimulation with enzymes and vermicompost (factor C) and that showed the highest removal percentage at 49 days (factor A) were included. These tests were selected because they were significant in the ANOVA. Therefore, tests 7 and 5 for the evaluation of factors A and B without considering the presence of factor C, and tests 14 and 15 evaluating the interaction of factors A, B, and C were analyzed by FTIR, contrasting each one with a blank soil sample (uncontaminated with automotive residual oil). The spectra are shown in Figure 1.

Functional groups such as alkanes in a wave number range from 2820 to 340 cm^−1^, alkynes in a wave number range from 2300 to 2420 cm^−1^, alkenes in a wave number range from 1660 to 1770 cm^−1^, and cycloalkenes in a wave number range from 1360 to 1480 cm^−1^ could be identified [44,45]. Alkanes, alkynes, alkenes, and cycloalkenes have been linked to heavy-phase hydrocarbons and residual lubricating oils [1,20,46,47].

The present study, in comparison with the optimal results of ambient and ideal conditions for the enzyme without and with the addition of vermicompost, showed favorable results in the degradation of the functional groups associated with heavy-phase hydrocarbons of the automotive residual oil. In Figure 1a, a transmittance of alkanes of 84% and cycloalkenes of 96.7% is shown; for these evaluated conditions, vermicompost was not added, nor were the ideal conditions for the enzyme presented, while for the Figure 1b–d, transmittances of alkanes and cycloalkenes of 98.86% and 98.1%, 98.7% and 98.4%, and 98.8% and 98.82% were obtained, respectively. On the other hand, Figure 1a shows transmittances of 99.4% and 99.2% for alkynes and alkenes, respectively. However, Figure 1b–d show a little noticeable transmittance for alkynes and alkenes, which is like the blank of the soil sample without the presence of automotive residual oil, suggesting the degradation of these compounds.

The degradation shown by the FTIR (Figure 1) follows a degradation trend very similar to that reported by Aguilera et al. [20], where the removal of automotive residual oil from contaminated soils was carried out using *Ricinus communis* L. enzymes at ideal conditions (temperature 37 °C and pH 4.5) in 49 days, obtaining a percentage of transmittance of alkanes of 97.6% and cycloalkenes of 99.6%, as well as the removal of alkynes and alkenes. This behavior can be seen qualitatively reflected in the presence of the mentioned functional groups associated with the hydrocarbons of automotive residual oil. Therefore, it can be suggested that biostimulation with enzymes present in *Ricinus communis* L. and the addition of vermicompost favors the degradation of the functional groups (heavy-phase hydrocarbons) present in the automotive residual oil, supporting the results obtained from the ANOVA (Table 4) for the significant factors that affect the response in the removal of automotive residual oil, being the most efficient treatment shown in Figure 1d.

### 3.5. Comparison with Other Studies 

Table 5 shows some technologies used for the remediation of hydrocarbon-contaminated soils performed under different conditions, highlighting the use of surfactants, enzymes, and vermicompost and comparing the results of this study. According to the results obtained from the experimental design by the ANOVA on the significant factors (Table 4) and the FTIR results shown in Figure 1, the compared bioremediation tests were 5, 7, 14, and 15, highlighting that test 14 obtained the highest removal efficiency of automotive residual oil from soil (Table 3).

It can be noted that the highest removal efficiency was obtained in this study for test 14 (Table 5) when *Ricinus communis* L. enzymes and vermicompost were used in the bioremediation process. Although Aguilera et al. [20] also reported a high efficiency using *Ricinus communis* L. enzymes (Table 5), this efficiency was enhanced when vermicompost was used as an amendment, as was done in this study. Mohammadi et al. [49] used vermicompost at different proportions. However, they obtained removal percentages 10% lower than this study for test 14, indicating that adding *Ricinus communis* L. enzymes was also essential to reach effective remediation. Likewise, the studies reported by Liu et al. [48] and Li et al. [50], employing persulfate and Triton X-100, showed efficiencies between 10 and 20% lower than this study for test 14 (Table 5). Therefore, biostimulation with *Ricinus communis* L. enzymes and vermicompost suggests that this technique could be a remediation technology with the highest removal efficiency of automotive residual oil in contaminated soils compared with other technologies. However, this process could still be optimized through an optimal design of response surface experiments evaluating factors such as enzyme extract concentration, vermicompost concentration, treatment time, and automotive residual oil concentration, among other parameters.

## 4. Conclusions

This study confirms the possibility of applying biostimulation with *Ricinus communis* L. enzymes and vermicompost (3% *w*/*v*–5% *w*/*w*) under the conditions of pH 4.5 and 37 °C (ideal for the highest catalytic activity of the enzymes) as an ex situ bioremediation technology of automotive residual oil-contaminated soils. The results showed an almost complete removal of the contaminant (99.90%) for 49 days of treatment with an initial concentration of 10,000 mg of automotive residual oil per kg of soil. According to the experimental design and ANOVA results, the biostimulation with enzymes and surfactant Triton X-100 was not significant. Therefore, the use of surfactant does not enhance the bioremediation process. The results of the present investigation open a new panorama of possible optimization in the bioremediation of automotive residual oil-contaminated soils by biostimulation with vermicompost and *Ricinus communis* L. enzymes.

## Figures and Tables

**Table 1:** Experimental design of 2^4^, used for the bioremediation tests.

Test	Time (Days)	pH and Temperature Conditions ^1^	Biostimulation with Enzyme and Vermicompost ^2^	Biostimulation with Enzyme and Surfactant ^3^
1	14	Ideal	Absent	Absent
2	14	Ideal	Absent	Absent
3	14	Ambient	Absent	Absent
4	14	Ambient	Absent	Absent
5	49	Ideal	Absent	Absent
6	49	Ideal	Absent	Absent
7	49	Ambient	Absent	Absent
8	49	Ambient	Absent	Absent
9	14	Ideal	Present	Absent
10	14	Ideal	Present	Absent
11	14	Ambient	Present	Absent
12	14	Ambient	Present	Absent
13	49	Ideal	Present	Absent
14	49	Ideal	Present	Absent
15	49	Ambient	Present	Absent
16	49	Ambient	Present	Absent
17	14	Ideal	Absent	Present
18	14	Ideal	Absent	Present
19	14	Ambient	Absent	Present
20	14	Ambient	Absent	Present
21	49	Ideal	Absent	Present
22	49	Ideal	Absent	Present
23	49	Ambient	Absent	Present
24	49	Ambient	Absent	Present
25	14	Ideal	Present	Present
26	14	Ideal	Present	Present
27	14	Ambient	Present	Present
28	14	Ambient	Present	Present
29	49	Ideal	Present	Present
30	49	Ideal	Present	Present
31	49	Ambient	Present	Present
32	49	Ambient	Present	Present

^1^ Ideal condition: soil pH modified to 4.5, temperature 37 °C. Ambient condition: soil pH without modification, room temperature. ^2^ Absent: without the presence of vermicompost. Present: with vermicompost. ^3^ Absent: without the presence of surfactant. Present: with surfactant.

**Table 2:** Physical-chemical parameters of the soil sampled and used in the bioremediation tests.

Parameter		Value	Unit
pH		7.01	-
Bulk density		1.45	g/mL
Moisture retention		41.54	%
Organic matter content		0.04	%
Inorganic nitrogen content		7.00	mg/kg
C/N ratio		57.1	-
Texture	Clay content	14.92	%
	Silt content	16.00	%
	Sand content	69.08	%

**Table 3:** Percentages of automotive residual oil removal obtained in bioremediation tests.

Test	Automotive Residual Oil Removal (%)	Test	Automotive Residual Oil Removal (%)
1	18.68	17	16.02
2	8.82	18	16.22
3	2.50	19	8.45
4	6.51	20	6.91
5	94.38	21	87.75
6	94.30	22	88.57
7	17.78	23	72.15
8	13.44	24	74.51
9	28.24	25	29.81
10	28.72	26	30.83
11	13.79	27	13.96
12	13.98	28	13.34
13	99.90	29	98.57
14	99.90	30	99.19
15	78.67	31	86.71
16	78.58	32	83.43

**Table 4:** Results of the analysis of variance (ANOVA) for automotive residual oil removal in soil.

Source	Sum of Squares	Degrees of Freedom	Mean Square	F Value	*p*-Value Prob > F	Significance
Model	38,689.55	4	9672.39	52.62	<0.0001	Significant
Factor A	31,944.44	1	31,944.44	173.80	<0.0001	Significant
Factor B	3942.50	1	3942.50	21.45	<0.0001	Significant
Factor C	2288.77	1	2288.77	12.45	0.0015	Significant
Factor D	513.84	1	513.84	2.80	0.1061	Not significant
Residual	4962.59	27	183.80			
Total	43,652.13	31				

Factor A: Time; factor B: pH and temperature conditions; factor C: biostimulation with enzyme and vermicompost; factor D: biostimulation with enzyme and surfactant.

**Table 5:** Comparison of remediation technologies with this study.

Remediation Technology	Treatment Conditions	Automotive Residual Oil Concentration (mg/kg)	Treatment Time (Days)	Removal Efficiency (%)	Reference
Biostimulation with *Ricinus communis* L. enzymes at ambient conditions	Enzymes: 3% *w*/*v* pH: 7.01 Temperature: room temperature	10,000	49	17.78	This study (Test 7)
Biostimulation with *Ricinus communis* L. enzymes and vermicompost at ambient conditions	Enzymes: 3% *w*/*v* Vermicompost: 5% *w*/*w* pH: 7.01 Temperature: room temperature	10,000	49	78.67	This study (Test 15)
Biostimulation with *Ricinus communis* L. enzymes at ideal conditions	Enzymes: 3% *w*/*v* pH: 4.5 Temperature: 37 °C	10,000	49	94.38	This study (Test 5)
Biostimulation with *Ricinus communis* L. enzymes and vermicompost at ideal conditions	Enzymes: 3% *w*/*v* Vermicompost: 5% *w*/*w* pH: 4.5 Temperature: 37 °C	10,000	49	99.90	This study (Test 14)
Bioremediation with *Ricinus communis* L. enzymes	Enzymes: 3% *w*/*v* pH: 4.5 Temperature: 37 °C	10,000	49	94.3	[20]
Remediation by persulfate oxidation coupled with microbial degradation	Persulfate dose: 1% Mixed bacteria used: *Enterobacteriaceae*, *Stenotrophomonas*, *Pseudomonas*, *Acinetobacter*, and *Achromobacter*	12,835	187	80.05	[48]
Bioremediation using vermicompost	Vermicompost: 2, 4, and 6% *w*/*w*	100, 200, and 300	45	83.00–89.00	[49]
Remediation by Triton X-100 aided washing	Stirring: 2000 rpm Temperature: 37 °C pH: 5.7	2206	1	90.36	[50]

## Data Availability

The datasets generated and/or analyzed during the current study are available from the corresponding author upon reasonable request.
